# Impact of analysis of the sentinel lymph node by one-step nucleic acid amplification (OSNA) compared to conventional histopathology on axillary and systemic treatment: data from the Dutch nationwide cohort of breast cancer patients

**DOI:** 10.1007/s10549-023-07065-0

**Published:** 2023-07-27

**Authors:** Elisabeth R. M. van Haaren, Ingrid G. M. Poodt, Merel A. Spiekerman van Weezelenburg, James van Bastelaar, Alfred Janssen, Bart de Vries, Marc B. I. Lobbes, Lee H. Bouwman, Yvonne L. J. Vissers

**Affiliations:** 1 Department of Surgery, Zuyderland Medical Centre, Dr. H. Van Der Hoffplein 1, 6162BG Sittard-Geleen, The Netherlands; 2 Department of Pathology, Zuyderland Medical Centre, Sittard-Geleen, The Netherlands; 3 Department of Radiology and Nuclear Medicine, Zuyderland Medical Center, Sittard-Geleen, The Netherlands; 4 GROW School for Oncology and Reproduction, Maastricht University, Maastricht, The Netherlands; 5 Department of Clinical Engineering, Faculty of Science and Engineering, Maastricht University, Maastricht, The Netherlands

**Keywords:** Breast cancer, Sentinel lymph node biopsy, One-step nucleic acid amplification, Adjuvant treatment

## Abstract

**Purpose:**

The outcome of the sentinel lymph node in breast cancer patients affects adjuvant treatment. Compared to conventional histopathology, analysis by one-step nucleic acid amplification (OSNA) harvests more micrometastasis, potentially inducing overtreatment. In this study we investigated the impact of OSNA analysis on adjuvant treatment, compared to histopathological analysis.

**Methods:**

Data from T1–3 breast cancer patients with sentinel nodes analysed between January 2016 and December 2019 by OSNA (OSNA group, *n* = 1086) from Zuyderland Medical Centre, the Netherlands, were compared to concurrent data from the Netherlands Cancer Registry (NKR) where sentinel nodes were examined by histology (histology group, n = 35,143). Primary outcomes were micro- or macrometastasis, axillary treatments (axillary lymph node dissection (ALND) or axillary radiotherapy (ART)), chemotherapy, and endocrine therapy. Statistics with Pearson Chi-square.

**Results:**

In the OSNA group more micrometastasis (14.9%) were detected compared to the histology group (7.9%, *p* < 0.001). No difference in axillary treatment between groups was detected (14.3 vs. 14.4%). In case of mastectomy and macrometastasis, ALND was preferred over ART in the OSNA group (14.9%) compared to the histology group (4.4%, *p* < 0.001). In cases of micrometastasis, no difference was seen. There was no difference in administration of adjuvant chemotherapy between groups. Endocrine treatment was administrated less often in the OSNA group compared to the histology group (45.8% vs. 50.8%, *p* < 0.002).

**Conclusion:**

More micrometastasis were detected by OSNA compared to histopathology, but no subsequent increase in adjuvant axillary and systematic treatment was noticed. When performing mastectomy and OSNA, there was a preference for ALND compared to ART.

## Introduction

Performing a sentinel lymph node biopsy in clinically node-negative breast cancer patients is still common practice for staging the axilla [1]. The occurrence of axillary metastasis influences the indication for adjuvant axillary treatment, i.e. axillary lymph node dissection (ALND) and axillary radiation therapy (ART). Indication for adjuvant systemic therapy is not only determined by patients and clinical-pathological characteristics [2] but also by the axillary nodal status [1]. 

To examine the sentinel node in breast cancer patients, conventional histopathological examination, using haematoxylin and eosin staining (H&E), in combination with immunohistochemical staining, is the most used method. [3] However, due to multilevel sectioning, this technique leads to significant tissue loss, therefore possible sampling error and consequently compromising accuracy. Moreover, these results could be further negatively influenced by interobserver variability. Another method for analysing the sentinel lymph node is one-step nucleic acid amplification (OSNA), an automated and reliable technique, analysing the complete lymph node, based on the measurement of mRNA of cytokeratin 19. [4, 5] Due to its relatively short analysis time, OSNA has the possibility to yield a direct intraoperative result without compromising sensitivity, a well-known disadvantage of other intraoperative methods, such as fresh frozen section analysis and imprint cytology. [6–9]

With the results of the American College of Surgeons Oncology Group (ACOSOG)-Z00-11 trial [10, 11], showing no benefit of an axillary lymph node dissection in T1–2 breast cancer patients with up to 2 positive nodes undergoing breast-conserving therapy, performing an intraoperatively OSNA could be debated. However, axillary radiation in case of low nodal involvement is still under debate. Moreover, mastectomy patients were not included and an intraoperative evaluation of the sentinel node could be valuable in these patients. Rubio et al. [12] described an overall concordance between conventional histology and OSNA of 96%. However, several other studies [13–15] pointed out that OSNA detected more micrometastasis. OSNA may be more accurate because it includes examination of the whole node instead of only a selection of slices of the node such as in histopathology. Since adjuvant treatments are to some extent guided by the finding of axillary metastasis a higher metastasis detection rate using OSNA potentially introduces overtreatment of patients with regards to adjuvant therapy, axillary lymph node dissection or axillary radiotherapy, and adjuvant systemic therapy. [16] The OSNA technique is favoured in several countries such as in Europe and in Japan and Australia, but not common practice in the USA or the Netherlands. Our institute implemented the OSNA method as standard practice for staging the sentinel lymph node in 2015.

In our previously published single-centre observational study [13], we detected more micrometastasis in the OSNA group when compared to the conventional histology group, but no difference was seen in administration of adjuvant treatment. However, this study was a single-centre study using a historical control group, this could be considered a bias. To address this matter, we compared the outcomes of sentinel lymph node biopsy analysed by OSNA to conventional histology analysis from data retrieved from the nationwide database of the Netherlands Cancer Registry (NKR) and studied the impact of the type of sentinel node analysis (OSNA or standard histology) on adjuvant axillary and systemic treatment in patients with primary clinically node-negative T1–3 breast cancer.

## Material and methods

### Patient selection and data collection

**Fig. 1:**
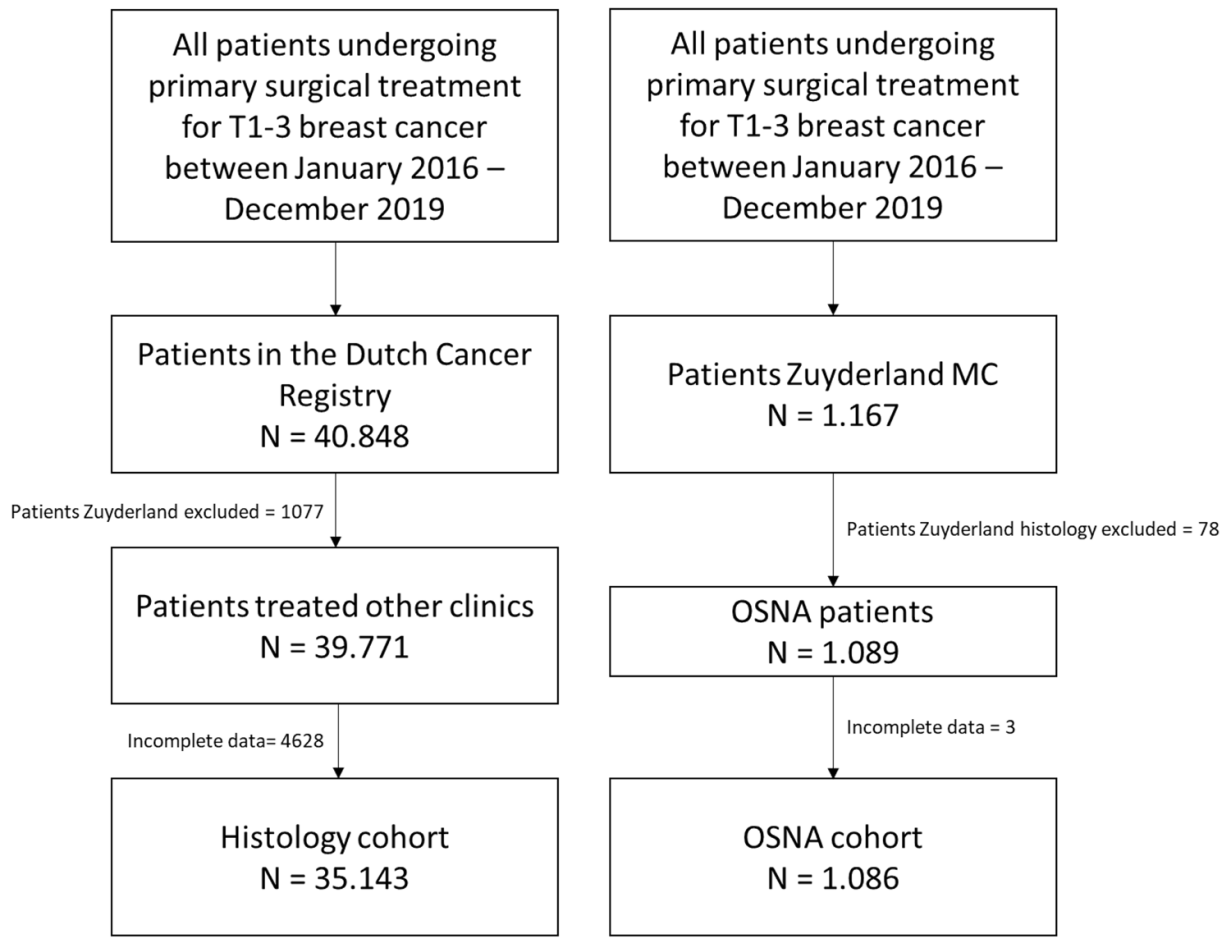
Flowchart patient selection and data collection

All data from patients with clinically node-negative T1–3 breast cancer undergoing primary surgical treatment and in whom sentinel node biopsy was performed between January 2016 and December 2019 were collected from the database of the Netherlands Cancer Registry (NKR), which is controlled by the Netherlands Comprehensive Cancer Organization (IKNL). Demographics regarding gender, age, tumour size, year of treatment, type of surgery, outcome of the sentinel lymph node biopsy (i.e. benign, micrometastasis or macrometastasis), axillary lymph node dissection, axillary radiotherapy, and adjuvant systemic therapy, i.e. chemotherapy, endocrine therapy, and chemo/immunotherapy, were extracted. In the Netherlands, Zuyderland Medical Centre is the only institution where sentinel lymph nodes biopsies are analysed using the OSNA technique. In all other Dutch hospitals, sentinel nodes are analysed by conventional histopathological technique. As the data from the Netherlands Cancer Registry does not include information on the specific method of analysis per sentinel node, we used the variable “institution” to create two groups: all data from Zuyderland Medical Centre were excluded, and all other data were assigned to the conventional histology group. Data were then crosschecked with the Zuyderland database of sentinel node biopsies performed by OSNA technique in the same period. Flowchart is shown in Fig. 1. We divided the data in two different surgical groups, i.e. breast-conserving treatment and mastectomy. In the Netherlands Cancer Registry, the amount of harvested sentinel lymph nodes per patient was not registered, only the concluding result was noted, i.e. benign, micrometastasis, or macrometastasis, the presence of isolated tumour cells was coded as benign. The results of our own database were coded in the same way, i.e. benign, micrometastasis, or macrometastasis.

Results are reported using STROBE statement guidelines [17].

### Histopathological examination and outcome of the sentinel lymph node biopsy

In the histology group, sentinel nodes were evaluated using standard conventional pathohistological examination. In this procedure, sentinel lymph nodes smaller than 5 mm were completely embedded, if not than embedded after slicing. After multilevel sectioning, haematoxylin and eosin staining was performed with additional immunohistochemical staining according to the Dutch breast cancer guidelines [18]. Lymph nodes were sliced in half along the long axis with size of 10 mm after formalin fixation. One part of the node was stained with haematoxylin and eosin and staged accordingly to the American Society of Clinical Oncology. [1, 19] The results of these sentinel nodes analysis were noted as benign, as micrometastasis (0.2-2mm) and as macrometastasis (> 2 mm). Isolated tumour cells (< 0.2 mm) were considered as a benign result. [18]

### OSNA technique and outcome of the sentinel lymph node biopsy

In the OSNA group, sentinel nodes were evaluated by the OSNA technique as described by Tsujimoto et al. [5]. This molecular technique quantifies the mRNA of cytokeratin 19 (CK19), an epithelial and nodal tumour marker. After surgical removal of the whole lymph node, the node was sent to the pathology department as a fresh specimen on ice. After removing the fatty tissue, the node was homogenised with 4-mL lysis buffer and centrifuged, after which a 2-µL sample was analysed in an automated gene amplification detection system using a reverse transcription loop-mediated isothermal amplification method with RT-LAMP (RD-100i system). The degree of amplification was detected via a by-product of the reaction and correlated to the number of CK19 mRNA copies per µL using a standard curve. The OSNA copy numbers were converted to standard histological measures for lymph node metastasis according to Tsujimoto et al. [5] as follows: < 2.5 × 10^2^ copies/µL of CK19 mRNA corresponds with a benign result, 2.5 × 10^2^–5 × 10^3^ copies/µL corresponds with micrometastasis, and > 5 × 10^3^ copies/µL corresponds with macrometastasis.

### Adjuvant systemic treatment

Primary outcome was the number of axillary treatments, divided in axillary lymph node dissection or axillary radiotherapy. In addition, the total number of patients receiving adjuvant systemic treatment was noted and divided into three groups. Patients receiving chemotherapy as monotherapy or a combination with (targeted) immunotherapy were allocated to the chemotherapy group, patients receiving a combination of chemotherapy and endocrine treatment were assigned to chemotherapy and endocrine group, and patients receiving only endocrine treatment were assigned to the endocrine group. The indications for systemic treatment are defined in the Dutch guidelines [20] and guided by the primary tumour characteristics (size, grade, receptor status) and nodal status. In these guidelines, micrometastasis are considered as node-positive results and therefore can influence the indication for adjuvant systemic treatment.

### Adjuvant locoregional treatment

According to Dutch guidelines [21], axillary therapy can be omitted in patients with micrometastasis undergoing breast-conserving therapy with whole breast radiation who receive adjuvant systemic treatment. However, if systemic therapy is skipped or risk factors are present, i.e. grade 3, lymphovascular invasion, triple negativity, tumour > 4 cm, or age < 40 years, adjuvant axillary radiation therapy is advised. In patients with nodal metastasis undergoing mastectomy, Dutch guidelines recommended axillary lymph node dissection for macrometastasis. Axillary radiotherapy is described as valid alternative. For micrometastasis, axillary treatment is only advised in case of risk factors, i.e. grade 3, lymphovascular invasion, triple negative, tumour > 4 cm, or age < 40 years.

### Statistical analysis

All data were described as means and standard deviations for continuous data. Categorical variables were noted as absolute numbers and percentages. Missing data were treated as such. Data analysis was performed with SPSS version 21.0 (IBM, NY, Unites States). Statistical significance was tested using Pearson Chi-square test for categorical variables. For continuous variables, independent sample *t* test was used. A univariable and multivariable logistic regression were performed to adjust for any statistically significant differences in baseline criteria. A two-sided *P* value < 0.05 was considered statistically significant.

## Results

Patients’ characteristics are summarised in Table 1. We included 35,143 patients in the histology group and 1086 patients in the OSNA group. Baseline parameters such as gender, tumour size, and year of diagnosis were comparable. There was a significant difference in age (range) with a higher percentage of patients of eighty years and older in the OSNA group, i.e. 11% versus 6.7% in the histology group (*p* < 0.001). The percentage of patients undergoing mastectomy was significantly higher in the OSNA group compared to the histology group, i.e. 34.9% versus 28.7% (*p* < 0.001) (Table 1).

**Table 1:** Patient characteristics

Parameter	Histology *n* = 35,143	%	OSNA *n* = 1086	%	*P-*value
Gender					0.246
Male	271	0.8	5	0.5	
Female	34,872	99.2	1081	99.5	
Age (range) in yrs	63 (20–96)		65 (29–92)		** < 0.001**
Age (yrs)					** < 0.001**
≤ 40	1044	3.0	25	2.3	
41–80	31,745	90.3	941	86.6	
≥ 80	2354	6.7	120	11.0	
cT stage					0.175
T1a/b	8940	25.4	295	27.2	
T1c	16,528	47.0	510	47.0	
T2	8407	23.9	234	21.5	
T3	658	1.9	21	1.9	
Tx	610	1.7	26	2.4	
Surgical treatment					** < 0.001**
Breast-conserving treatment	25,046	71.	711	65.5	
Mastectomy	10,097		375	34.9	
Year					0.323
2016	9050	25.8	304	28.0	
2017	9088	25.9	271	25.0	
2018	8625	24.5	269	24.8	
2019	8380	23.8	242	22.3	

Significant differences are bold

Statistical analysis was performed using independent sample *t* test for continuous and Pearson Chi-square test for categorical data

No difference in the percentage of patients with macrometastasis was observed between the OSNA and the histology group. We found significantly more patients with only micrometastasis in the OSNA group: 14.9% compared to the histology group 7.9% (*p* < 0.001). Results are shown in Table 2.

**Table 2:** Overall results of sentinel node analysis

Parameter	Histology *n* = 35,143	%	OSNA *n* = 1086	%	*P-*value
Macrometastasis	4449	12.7	116	10.7	0.053
Micrometastasis	2771	7.9	162	14.9	** < 0.001**
Axillary therapy*					0.083
Yes	5046	14.4	155	14.3	
No	29,937	85.2	931	85.7	
Unknown	160	0.5	0	0.0	
Axillary lymph node dissection	723	2.1	60	5.5	** < 0.001**
Axillary radiotherapy					** < 0.001**
Yes	4323	12.3	95	8.7	
No	30,660	87.2	991	91.3	
Unknown	160	0.5	0	0.0	
Chemotherapy**	6655	18.9	229	21.1	0.075
Endocrine therapy	17,852	50.8	497	45.8	**0.001**
Chemotherapy and endocrine therapy	4873	13.9	133	12.2	0.128

Significant differences are bold

^*^Axillary lymph node dissection or axillary radiotherapy

**Chemotherapy with or without (targeted) immunotherapy. Statistical analysis was performed using Pearson Chi-square test

This latter finding was consistent in the mastectomy group: 15.5% in OSNA and in 10.4% histology (*p* < 0.002) and in the breast-conserving treatment group: 14.6% in OSNA versus 6.9% in histology (*p* < 0.001). Results are shown in Tables 3 and 4.

**Table 3:** Results of sentinel node analysis in Mastectomy

Parameter	Histology *n* = 10,097	%	OSNA *n* = 375	%	*P-*value
Macrometastasis	1927	19.1	65	17.3	0.396
Micrometastasis	1048	10.4	58	15.5	**0.002**
Axillary therapy*					0.591
Yes	1828	18.11	75	20.00	
No	8264	81.8	300	80.0	
Unknown	5	0.05	0	0.0	
Axillary lymph node dissection	445	4.4	56	14.9	** < 0.001**
Axillary radiotherapy					** < 0.001**
Yes	1383	13.7	19	5.1	
No	8709	86.3	356	94.9	
Unknown	5	0.05	0	0.0	
Chemotherapy**	2471	24.5	96	25.6	0.618
Endocrine therapy	6194	61.3	211	56.3	**0.048**
Chemotherapy and endocrine therapy	1900	18.8	66	17.6	0.553
cT stage					0.188
T1a/b	1539	15.2	61	16.3	
T1c	3826	37.9	150	40.0	
T2	3931	38.9	130	34.7	
T3	598	5.9	21	5.6	
Tx	203	2.0	13	3.5	

Significant differences are bold

^*^Axillary lymph node dissection or axillary radiotherapy

**Chemotherapy with or without (targeted) immunotherapy. Statistical analysis was performed using Pearson Chi-square test

**Table 4:** Results of sentinel node analysis in Breast-conserving therapy

Parameter	Histology *n* = 25,046	%	OSNA *n* = 711	%	*P-*value
Macrometastasis	2522	10.1	51	7.2	**0.011**
Micrometastasis	1723	6.9	104	14.6	** < 0.001**
Axillary therapy*					**0.046**
Yes	3218	12.8	80	11.3	
No	21,673	86.5	631	88.7	
Unknown	155	0.6	0	0.0	
Axillary lymph node dissection	278	1.1	4	0.6	0.167
Axillary radiotherapy					0.072
Yes	2940	11.7	76	10.7	
No	21,951	87.6	635	89.3	
Unknown	155	0.6	0	0.0	
Chemotherapy**	4184	16.7	133	18.7	0.159
Endocrine therapy	11658	46.5	286	40.2	**0.001**
Chemotherapy and endocrine therapy	2973	11.9	67	9.4	**0.046**
cT stage					0.072
T1a/b	7401	29.6	234	32.9	
T1c	12,702	50.7	360	50.6	
T2	4476	17.8	104	14.6	
T3	60	0.2	0	0.0	
Tx	407	1.6	13	1.8	

Significant differences are bold

^*^Axillary lymph node dissection or axillary radiotherapy

**Chemotherapy with or without (targeted) immunotherapy. Statistical analysis was performed using Pearson Chi-square test

A total of 5201 patients received axillary treatment, i.e. axillary lymph node dissection or axillary radiotherapy, showing no significant difference between the histology (14.4%) and the OSNA (14.3%) group. In the OSNA group, the percentage of axillary lymph node dissections was significantly higher (5.5%) than in the histology group (2.1%) and axillary radiotherapy (8.7%) significantly lower than in the histology group (12.3%) (both *p* < 0.001) results presented in Table 2.

**Fig. 2:**
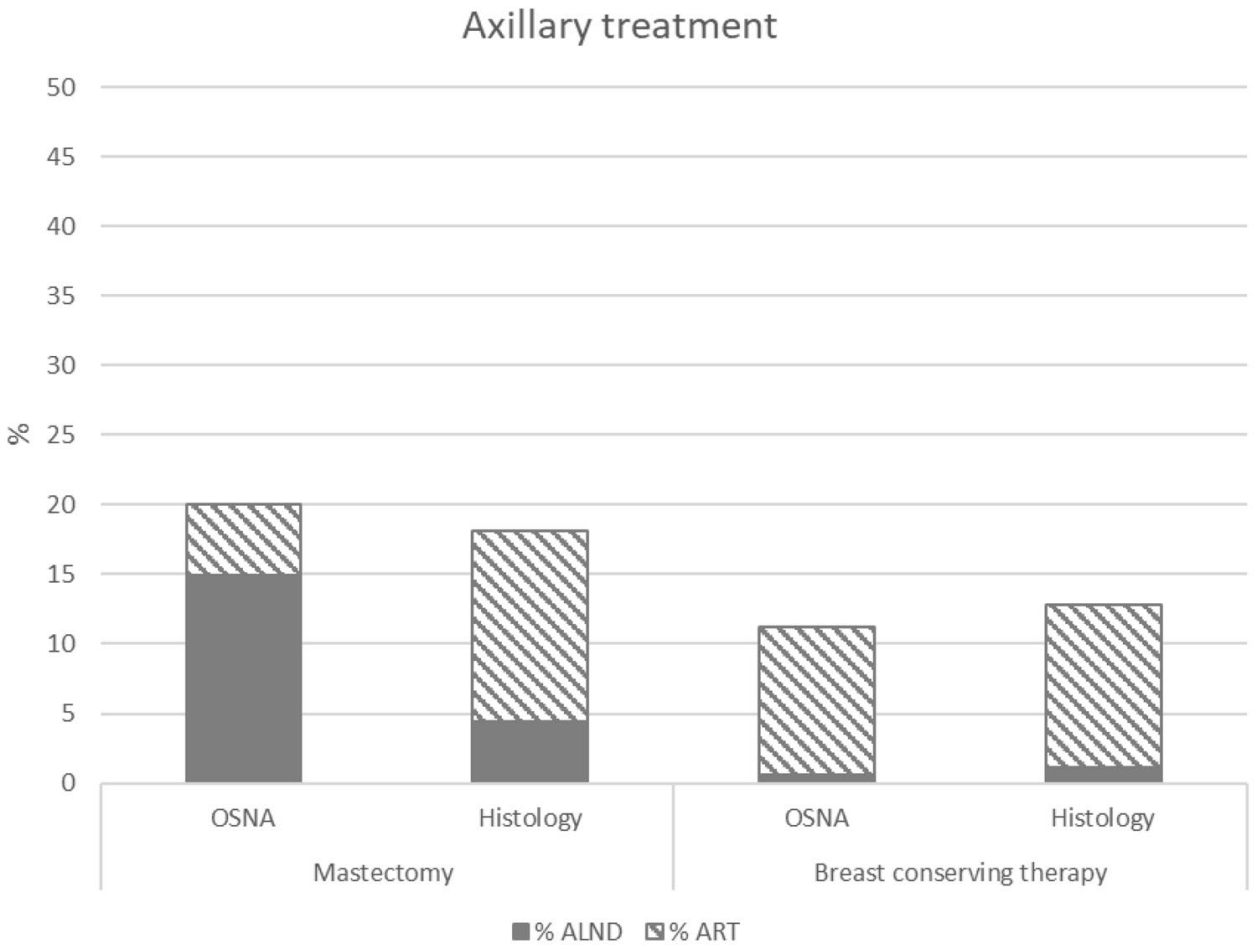
Axillary treatment in mastectomy or breast-conserving treatment

In the patients undergoing a mastectomy and OSNA, 14.9% underwent an axillary lymph node dissection versus 4.4% in the histology group (*p* < 0.001) and axillary radiotherapy was given in 5.1% of the patients in the OSNA group versus 13.7% in the histology group (*p* < 0.001), without difference in total adjuvant axillary therapy. No significant difference was observed in the patients undergoing breast-conserving therapy shown in Fig. 2.

**Fig. 3:**
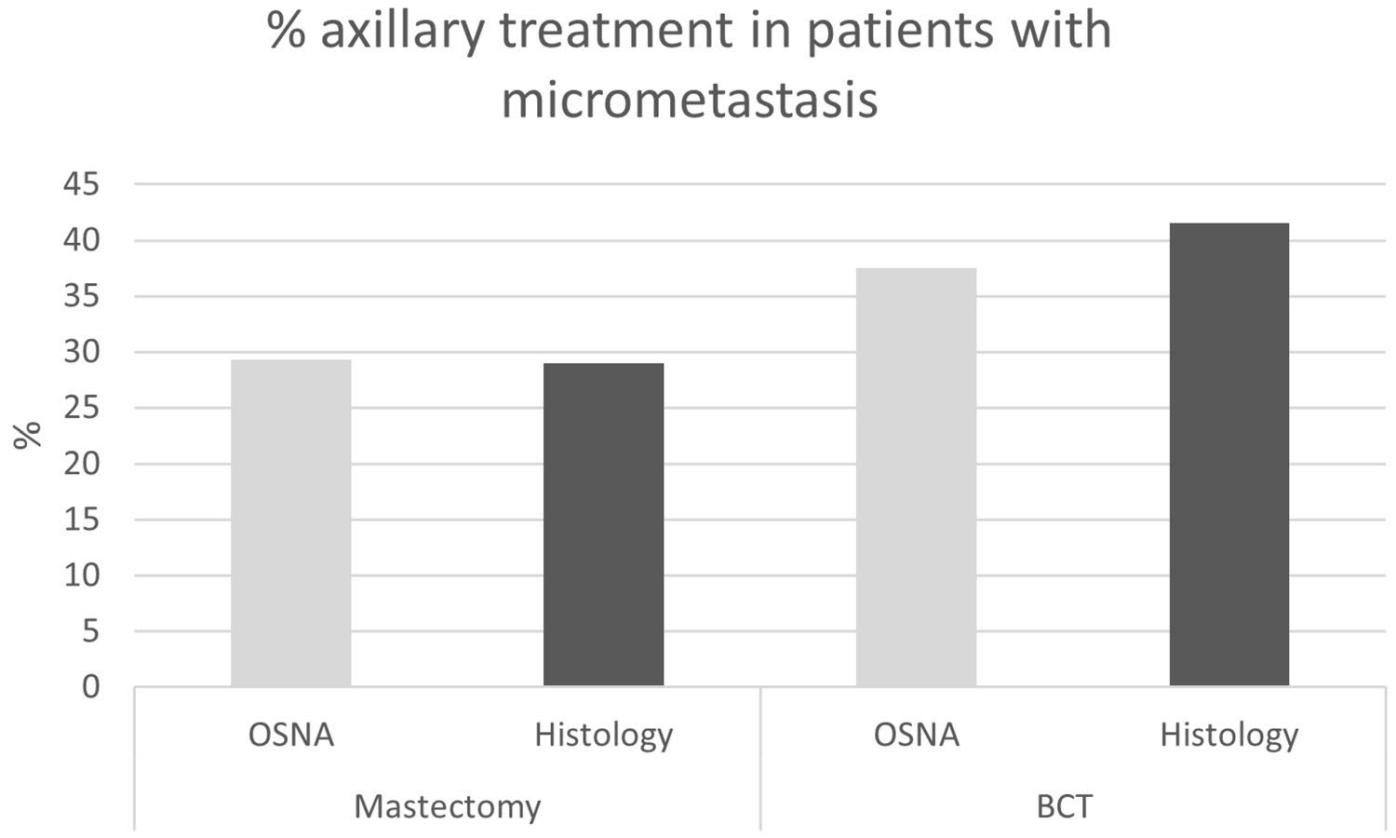
Percentage axillary treatment in patients with micrometastasis

In case of patients undergoing a mastectomy and showing a macrometastasis, ALND was performed 14.9% in the OSNA group when compared to 4.4% in the histology group (*p* < 0.001). In case of a micrometastasis and breast-conserving therapy, 37.5% in the OSNA group compared to 41.5% in the histology group received axillary treatment as shown in Fig. 3. In case of mastectomy and micrometastasis, this was 29.3% in the OSNA and 29.0% in the histology group.

The results of adjuvant systemic treatment including chemotherapy and chemotherapy in combination with endocrine treatment showed no overall difference between the histology and OSNA group (Table 2). The percentage of patients receiving only endocrine treatment was significantly lower in the OSNA group 45.8% compared to the histology group 50.8% (*p* < 0.002). These results were consistent in the mastectomy and breast-conserving treatment group as shown in Tables 3 and 4. In the breast-conserving treatment group, there was a significant difference in the combination chemotherapy with endocrine treatment between the OSNA group (9.4%) and histology group (11.9%) *p* < 0.046 (Table 4).

To rule out bias from the possible confounders’ age and surgical procedure, a univariable and multivariable logistic regression was performed as shown in Table 5. The adjusted OR show no significant difference on axillary or adjuvant systemic treatment between OSNA and histology.

**Table 5:** Unadjusted and adjusted odds ratios for axillary treatment and adjuvant systemic treatment (adjusted for age and surgical procedure)

	Unadjusted OR (95% CI)	*p*-value	Adjusted OR (95% CI)	*p*-value
Axillary treatment	0.988 (0.831–1.174)	0.889	1.007 (0.846–1.197)	0.939
Adjuvant systemic treatment	1.054 (0.933–1.189)	0.399	1.035 (0.915–1.172)	0.585

## Discussion

Omitting the sentinel lymph node biopsy in clinically node-negative invasive breast cancer patients undergoing breast-conserving therapy is under investigation [22–24]; it is however still common practice to examine the sentinel node in patients undergoing breast-conserving therapy or mastectomy. Pathological nodal status affects the indication for adjuvant axillary and systemic treatment. To investigate the sentinel lymph node biopsy, conventional histopathological examination is the standard method worldwide, although multiple hospitals in Japan, Australia, and Europe currently use the OSNA technique. In this study, we compared the results from OSNA in our database to the histology data of the Netherlands Cancer Registry (NKR) and detected a significantly higher number of micrometastasis in the OSNA group, 14.9% versus 7.9%. These results were concordant with outcomes published by several authors. [13–15] In the literature there are contradicting results regarding the impact of micrometastasis on prognosis. Several studies [25–27] claimed that micrometastasis had no impact on disease-free and overall survival, but the study of Anderson [28] demonstrated a worse disease-free survival. Moreover, a better survival was confirmed in patients with micrometastasis who had received adjuvant therapy compared to having received no adjuvant treatment in the study of de Boer et al. [29]. These conflicting results address the ongoing debate on the prognostic value of micrometastatic node involvement and impact on adjuvant axillary treatment.

It is assumed that a higher detection rate of micrometastasis could lead to axillary overtreatment, i.e. more axillary lymph node dissection or axillary radiotherapy, a potential drawback in the use of the OSNA method. The overall results, however, did not show any significant difference in axillary treatment between the OSNA (14.3%) and the histology group (14.4%) (Table 2). Incorporating the results of the American College of Surgeons Oncology Group (ACOSOG) Z0011 trial and the International Breast Cancer Study Group (IBCSG) 23-01 trial [30–32], indications for axillary treatment were changed and restricted to patients with extended tumour burden in the axilla and in this era it could be assumed that micrometastatic disease has no impact on adjuvant axillary treatment. However, according to the Dutch guidelines [21], in the Netherlands axillary therapy is omitted in patients with micrometastasis undergoing breast-conserving therapy with whole breast radiation who receive adjuvant systemic treatment, but if systemic therapy is skipped or risk factors are present, i.e. grade 3, lymphovascular invasion, triple negative, tumour > 4 cm, or age < 40 years, adjuvant axillary radiation therapy is considered. Results showed that in case of micrometastasis and breast-conserving therapy, 37.5% (OSNA) and 41.5% (histology) of the patients received axillary treatment. And Table 3 shows that axillary radiation therapy is favoured in cases undergoing breast-conserving therapy in the presence of any nodal metastasis.

More patients in the OSNA group underwent a mastectomy, the reason is uncertain, maybe influenced by personal preference or higher age. In patients undergoing mastectomy, who do not meet the Z0011 criteria, axillary lymph node dissection is still recommended if there is nodal metastasis, although doubtful in case of micrometastasis. Both ESMO, ASCO [1, 33], and national Dutch guidelines [21] describe axillary radiotherapy as valid alternative, based on the findings of the AMAROS trial [34, 35], that showed no significant difference in axillary recurrence and disease-free survival between axillary lymph node dissection and axillary radiotherapy in patients with a tumour positive sentinel node. Therefore, axillary lymph node dissection or axillary radiotherapy are applied interchangeably depending on hospital and patient preferences. In our study, we demonstrated no overall difference in axillary treatment in patients undergoing mastectomy. However, in the histology group, axillary radiotherapy was favoured, and in the OSNA group, there was a preference for axillary lymph node dissection. Since the OSNA technique is an automated assessment with a short examination time (30 min) offering a direct intraoperative result, it has the possible advantage to execute an axillary lymph node dissection in the same procedure. This prevents patients having to undergo second surgical procedure but also makes axillary adjuvant radiotherapy postoperatively in multiple fractions unnecessary. In the histology group, results of the sentinel node biopsy are only available after several days, thereby delaying axillary lymph node dissection and requiring a secondary procedure. This could be conceived as a burden for patients. In Zuyderland Medical Centre, all patients are counselled preoperatively about possible adjuvant axillary therapies in case of a positive sentinel node. In our experience, patients who prefer axillary lymph node dissection above axillary radiotherapy often consider it as an advantage to undergo immediate axillary surgical treatment and accept the higher risk of developing oedema.

The indication for adjuvant systemic treatment in clinically node-negative breast cancer patients is not only based on patients’ characteristics such as age, menopausal status, morbidity, and tumour biology, such as size, grade, and receptor status, but also on the presence of nodal metastases. The presumption that finding more micrometastasis when applying OSNA would lead to a higher number of patients receiving adjuvant systemic therapy was not confirmed in our study: no significant difference in overall systemic treatment between the chemotherapy and combination with endocrine therapy was found, independent of the surgical intervention. The reason why less endocrine treatment was given in the OSNA group when compared to the histology group remains unclear. Hypothetically speaking, the higher percentage of patients > 80 years in the OSNA group could have led to more declining of adjuvant endocrine treatment, in line with the literature showing that elderly patients tend to receive suboptimal adjuvant treatment [36–38].

Although mentioned in literature [28, 29] that micrometastasis could be associated with worse prognosis and patients should be treated accordantly, this study collects solid evidence from a large database that the presence of more micrometastasis with the OSNA technique did not lead to the institution of more adjuvant therapy. Long-term results on recurrence or survival of patients with micrometastasis were not the scope of our research but could be of future interest.

Strengths of the current findings lie in the large cohort of patients, in a broad timeline. The independent national register of data of the Netherlands Cancer Registry was used, supporting objective, unbiased, and reliable collection of data from all hospitals in the Netherlands. To date, this is the largest study focussing on the consequences of various analytic strategies for the sentinel node on adjuvant therapies. Many publications regarding OSNA have focused on its capabilities in risk stratification for non-sentinel node involvement. However, studies reporting on the oncological consequences of OSNA are scarce. A recent paper from Bertozzi et al. [14] compared survival data between different methods of nodal staging, i.e. OSNA, frozen section, and histology. In accordance with our results, they also found a higher amount of micrometastasis when using OSNA when compared to histology. Nevertheless, they demonstrated similar overall and disease-free survival. Our study thus contributes to the body of evidence of real-world oncological data supporting further adoption of OSNA as a routine technique to investigate the sentinel lymph node biopsy.

One of the limitations of this study was the high discrepancy in number of patients per group, although we believe that by maintaining strict inclusion criteria and after statistical testing for bias the two cohorts are reliably comparable.

Moreover, the disproportionate distribution of hospitals using OSNA, only one institution in the Netherlands, and consequently, the bias due to regional differences in preferences for performing axillary lymph node dissection or axillary radiotherapy. However, since the indications for adjuvant systemic or locoregional treatments are strictly defined in the Dutch national guidelines for breast cancer, practices of all centres are comparable with respect to the indication of adjuvant treatment. We therefore think that it is justified to ascribe our outcomes to the found differences in the amount of micrometastasis (15 versus 8%) and not to varying practices amongst centres.

## Conclusion

Evaluating OSNA and histology data from the nationwide cohort of the Netherlands Cancer Registry (NKR) showed that in clinically node-negative T1–3 breast cancer patients, the sentinel lymph node biopsy analysed by the one-step nucleic acid amplification technique showed more micrometastasis when compared to conventional histopathological examination. Although speculated this could lead to overtreatment, no escalation in administrating adjuvant locoregional and systemic treatment was detected.

## Data Availability

The datasets analysed during this current study are not publicly available but are available from the corresponding author on reasonable request. The datasets are collected out of our own database and collected of the database of the Netherlands Cancer Registry (NKR). The authors thank the registration team of the Netherlands Comprehensive Cancer Organization (IKNL) for the collection of data for the Netherlands Cancer Registry (IKNR*).*

## Abbreviations

OSNA: One-step nucleic acid amplification
ALND: Axillary lymph node dissection
ART: Axillary radiation therapy
